# Co-designing a theory-informed intervention to increase shared decision-making in maternity care

**DOI:** 10.1186/s12961-023-00959-x

**Published:** 2023-01-31

**Authors:** Alex Waddell, Gerri Spassova, Louise Sampson, Lena Jungbluth, Jennifer Dam, Peter Bragge

**Affiliations:** 1 Monash Sustainable Development Institute, Monash University, 8 Scenic Boulevard, Clayton Campus, VIC 3800 Clayton, Australia; 2 Victorian Department of Health, Safer Care Victoria, 50 Lonsdale St, Melbourne, VIC 3000 Australia; 3 Department of Marketing, Monash Business School, 900 Dandenong Rd, Caulfield East, Victoria, 3145 Australia; 4 Royal Women’s Hospital, 20 Flemington Rd, Parkville, Melbourne, VIC 3052 Australia; 5 Evidence Review Service, Monash Sustainable Development Institute, Monash University, 8 Scenic Boulevard, Clayton, VIC 3800 Australia

**Keywords:** Shared decision-making, Health policy research, Health service research, Maternity care, Co-design, Hospital accreditation

## Abstract

**Background:**

Shared decision-making (SDM) has been shown to improve healthcare outcomes and is a recognized right of patients. Policy requires health services to implement SDM. However, there is limited research into what interventions work and for what reasons. The aim of the study was to develop a series of interventions to increase the use of SDM in maternity care with stakeholders.

**Methods:**

Interventions to increase the use of SDM in the setting of pregnancy care were developed using Behaviour Change Wheel and Theoretical Domains Framework and building on findings of an in-depth qualitative study which were inductively analysed. Intervention development workshops involved co-design, with patients, clinicians, health service administrators and decision-makers, and government policy makers. Workshops focused on identifying viable SDM opportunities and tailoring interventions to the local context (the Royal Women’s Hospital) and salient qualitative themes.

**Results:**

Pain management options during labour were identified by participants as a high priority for application of SDM, and three interventions were developed including patient and clinician access to the Victorian Government’s maternity record via the patient portal and electronic medical records (EMR); a multi-layered persuasive communications campaign designed; and clinical champions and SDM simulation training. Factors identified by participants for successful implementation included having alignment with strategic direction of the service, support of leaders, using pre-standing resources and workflows, using clinical champions, and ensuring equity.

**Conclusion:**

Three interventions co-designed to increase the use of SDM for pain management during labour address key barriers and facilitators to SDM in maternity care. This study exemplifies how health services can use behavioural science and co-design principles to increase the use of SDM. Insights into the co-design of interventions to implement SDM in routine practice provide a framework for other health services, policy makers and researchers.

**Supplementary Information:**

The online version contains supplementary material available at 10.1186/s12961-023-00959-x.

## Introduction

Shared decision-making (SDM) is an active two-way process by which clinicians and patients discuss and decide on the next step in the patient’s care [1, 2]. In the SDM process, the patient is considered the expert in their own life, values and goals, while the clinician outlines risks and benefits of treatment options. Together, they discuss the options and weigh up the risks and benefits in line with the clinical evidence and patient preference [3, 4]. The key difference of SDM to other forms of decision-making, such as paternalism and informed consent, is the flow of information between clinician and patient. Under a paternalistic style of decision-making, the flow of information is from the clinician to the patient, with care done to the patient as a passive recipient [1, 5]. Under informed consent, patients are provided information of risks and benefits of treatment in line with legal and regulatory requirements to obtain consent [6, 7]. Informed consent is usually provided after a decision has been made about next steps in treatment and details only one treatment option [7, 8]. The SDM process allows for information to be shared between the patient and clinician to explore alternatives to the most common treatment options that may be preferred by the patient, based on their goals, preferences and values [6]. SDM has been shown to decrease healthcare utilization and care variation and increase patient-reported health outcomes, as well as communication between clinicians and patients [7–9]. Failure to include patients in decisions about their care has been shown to increase decisional conflict and patients’ feeling of being uninformed [10].

SDM is a practical process through which to provide patient-centred care [4, 11]. Patient-centred care treats the patient as a person rather than a set of signs or symptoms, understanding that risks and benefits change depending on the needs of the individual [9]. SDM embraces uncertainty by supporting patients’ autonomy and self-determination as an equal partner in decision-making [4, 12].

It is considered a right of all patients to be included in decisions about their care [13]. Therefore, in Australia, similarly to other nations [14], there are clear policy directives at both the state and federal level that require or mandate health services to implement SDM [15, 16]. The National Safety and Quality in Health Service (NSQHS) Standards explicitly mention SDM (e.g. Standards 2 and 5). Health services in Australia must provide evidence to prove the implementation of SDM throughout the service to meet accreditation requirements [16]. Specifically, accreditors are looking for evidence such as policy documents or processes for SDM, training documents, audits or observations, the involvement of clinicians and patients in decision-making, or analysis of feedback from staff and patients [16]. These policy directives underline that SDM should be expected by patients when accessing health services [17] and that health services must implement SDM throughout their service [16].

In the State of Victoria, as in other jurisdictions, SDM is still not routine practice [18, 19], with only 68% of patients reporting they have been included in decisions as much as they would have liked and only 55.9% reporting that staff spoke to them about their options [20]. As such there appears to be a gap between what health services are expected to do, as directed by policy, and what is happening in practice.

The Royal Women’s Hospital (the Women’s) is a large public maternity hospital. In line with the federal NSQHS Standards [16] and Victorian State policy directive, the Partnering in Healthcare Framework [15], the Women’s is required to implement SDM throughout their service. In addition to the federal and state policy drivers [15, 16], the Women’s makes explicit their intention to practise SDM through their mission statement and strategic plan.

However, tertiary health services such as the Women’s have been left with a complex problem – how to increase the use of SDM in their service when there is a lack of evidence for what works, why and how. The context of maternity care adds to this complexity with the majority of patients in Australia provided maternity care by large public health services (such as the Women’s) across outpatient and inpatient settings. The rights of the unborn foetus also add complexity with many patients seeing the foetus as another patient for whom decisions are made [21]. Patients in Victorian maternity care services report similar lack of inclusion in decisions about their care to the general population, with 24.4% of women experiencing a passive role in decision-making [19].

In maternity care settings there are numerous studies investigating patients’ and clinicians’ perceptions of decision-making for specific decisions [e.g. whether or not to have a caesarean section (CS) or vaginal birth after caesarean section (VBAC)] [22]. Interventions to address these tend to focus on increasing patient’s knowledge, with very few attempting to change clinicians’ behaviour [22]. Following a systematic review of SDM for CS, Coates et al. (2020) call for interventions address clinicians’ behaviours and to include both patients and clinicians in their development [22].

SDM research more broadly has predominantly occurred in primary and secondary care settings and tends to focus on patient and clinician barriers and facilitators to SDM, rather than organizational or systemic barriers and facilitators [23, 24]. Furthermore, much of the research to date lacks clear evidence for what facilitates the use of SDM, either due to unclear reporting or due to a lack of theory underpinning intervention design [25, 26].

Successful implementation relies on utilizing best-available evidence and incorporating the experiences of stakeholders [27], which can be applied to SDM [18]. For example, including stakeholders provides insight into the individual, organizational and system-level factors influencing SDM [23, 28, 29]. Furthermore, the experiences and expertise of these stakeholders may provide insight into what will and will not work, why and how within their own service, increasing the chances of success. Failure to include stakeholders may result in failed implementation efforts [18]. Behavioural science and co-design address these issues. Behavioural science is systematic and theory driven. It seeks to identify and assess the problem, develop possible interventions to solve the problem, and evaluate the interventions [30, 31]. Co-design, on the other hand, seeks to include end users and stakeholders in all aspects of research across the life cycle of research projects, including decision-making, design, monitoring and evaluation to facilitate meaningful and material involvement [32].

**Fig. 1:**
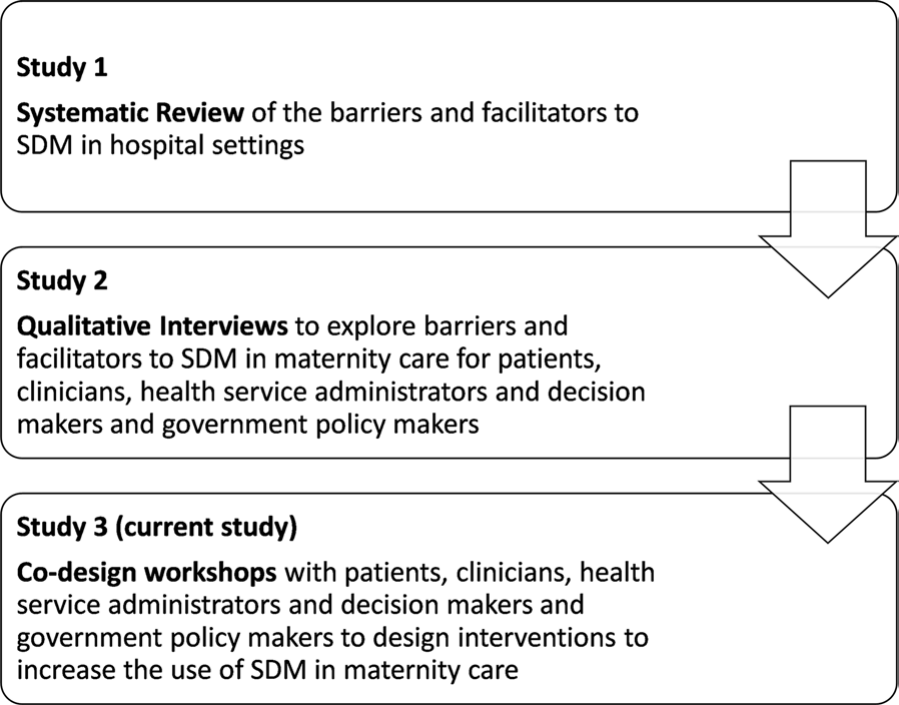
Overview of studies contributing to larger behavioural science research project

This study was the third phase in a larger research project, including a systematic review of the barriers and facilitators to SDM in hospital settings [24] and in-depth qualitative interviews with patients, clinicians, health service administrators and decision makers and government policy makers to understand their barriers and facilitators to SDM at the Women’s [33] (Fig. 1). Major barriers to SDM include lack of continuity of care, lack of knowledge from patients and clinicians, clinician’s lack of SDM skill, and policies and guidelines that do not support SDM. Major facilitators included changes to policy and guideline, increased knowledge and professional role factors and social influences. This study aimed to combined behavioural science and co-design to prioritize and develop a series of interventions to increase the use of SDM in maternity care at the Women’s.

## Methods

### Study design

**Fig. 2:**
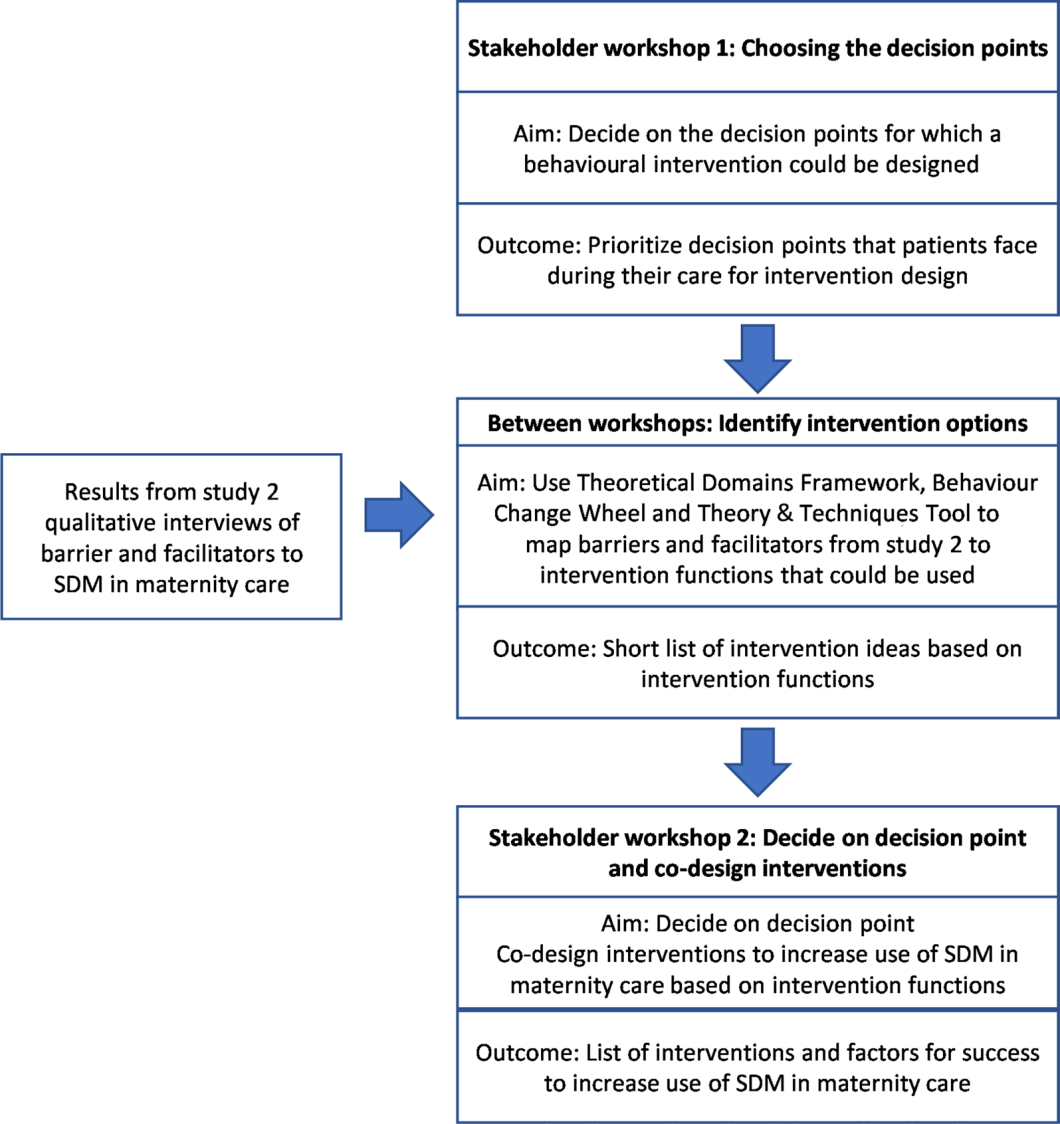
Overview of methods

This qualitative study involved two stakeholder co-design workshops. Through ‘collective making’, co-design workshops enable stakeholders to discover, share and combine their tacit knowledge and to create actionable interventions [34]. The current study built on results from previous studies within the larger behavioural science research project (Fig. 1). Study design, data collection and analysis are based on the Consolidated Criteria for Reporting Qualitative Research (COREQ) [35] (Additional file 1). Figure 2 summarizes the study design.

### Overview of development of the interventions

Behavioural science provides a framework for understanding the factors (barriers and facilitators) that influence actors and stakeholders, and for designing interventions to address these [36]. The behaviour change wheel (BCW)[37] is a guide for researchers and practitioners that synthesizes 19 frameworks of behaviour change to identify, understand and create interventions to change behaviour [37]. It is often used in conjunction with the theoretical domains framework (TDF) [36] to understand the barriers and facilitators to behaviour in more detail. Importantly, the BCW and TDF recognize that behaviour is influenced by a complex set of interconnected factors, and any intervention to change behaviour should aim to address one or more of these [31]. Together, the BCW and TDF take practitioners through steps of how to design interventions to influence behaviour based on barriers and facilitators to the behaviour. The BCW leaves much of the design of interventions open to the discretion of researchers, and each step relies on numerous decisions [38]. Research to date has not explicitly reported how researchers make decisions and move between steps; in this respect the BCW remains largely a ‘black box’ [38]. Likewise, the BCW does not detail how to involve stakeholders in the design of interventions. For this reason, co-design principles were employed [34].

### Sample and setting

This study took place at a large Australian public maternity service providing care to approximately 7000 patients and babies per year through pregnancy, birth and after birth. The Women’s provides care for all pregnancies, from uncomplicated low-risk health pregnancies to complex or high-risk pregnancies.

Workshop participants included patients, clinicians, health service administrators and decision makers, and government policy makers. Patients who had participated in the second phase of the research (qualitative interviews; Fig. 1) were invited to take part (supplementary material). Clinicians, health service administrators and decision makers, and government policy makers were recruited via snowball sampling [39] (Additional file 1). Prior to the workshops, participants were sent an ethics-approved briefing document outlining key information regarding SDM, study findings to date, the aims and agenda.

### Ethical considerations

Ethics approval was obtained from the Royal Women’s Hospital Human Research Ethics Committee (reference HREC/73706/RWH-21-14) prior to data collection. All participants provided informed consent.

### Data collection and analysis

Workshops were held via the online video platform Zoom in December 2021 and February 2022. They were facilitated by A.W. Data in the form of field notes, the contents of online whiteboards, and online voting programmes were collected from both stakeholder workshops. Field notes were taken by five researchers (A.W., P.B., L.J., J.D., L.S.) throughout each workshop [40]. Recordings of the workshops were not taken to encourage open and honest conversation.

Thematic analysis [41] was based on a ‘best fit framework synthesis’ (BFFS) [42] as recommended by The Cochrane Qualitative Review Methods Group [43]. The framework allows for synthesis to be based on previous published models, thereby building on pre-existing evidence with new knowledge [42]. The models used for analysis were the TDF, BCW and Theory & Techniques Tool (TATT) [31, 36, 37, 45]. Data were collated and analysed by one researcher who discussed all coding decisions with two researchers [40].

### Stakeholder workshop 1: choosing the decision points

The aim of the first workshop was to decide on potential SDM decision points in pregnancy. Workshop activities were based on ‘collective-making’, and used journey mapping and prioritization based on co-design principles [32, 34, 44]. A brief introductory presentation was provided to participants outlining the definition and practical use of SDM. After addressing participants’ questions, journey mapping was used to facilitate discussions about different decision points during maternity care. A list of potential decision points was generated and combined with study 2 results and presented to the group. Subsequently, participants used an electronic poll to independently and anonymously score the presented decision points on the basis of a range of factors (feasibility, clinical importance, patient benefit, clinician acceptability) [27] (Additional file 1). These factors were selected as they were broad enough for each participant to answer from their own perspective and to stimulate discussion. The results of the poll were shared with participants who further discussed what each factor meant for them and how it influenced their decision. Through discussion, two high-priority decision points for implementation of an SDM intervention at the Women’s were decided on. Participants were asked to consider which of the two decision points would be most appropriate and practical to implement within a 12-month time period. Those that participated in both workshops were asked to deliberate over the 16 weeks between workshops 1 and 2.

### Between workshops: identify intervention options

Between workshop 1 and workshop 2, A.W. in consultation with P.B. conducted analysis of workshop 1 data and results from study 2 using the TDF, BCW and Theory & Techniques Tool (TATT), [31, 36, 45, 47] as outlined below.

### Barriers and facilitators identified in study 2

In study 2, barriers and facilitators to SDM in maternity care were investigated using qualitative interviews (citation in progress). Importantly, these interviews were conducted with patients, clinicians, health service administrators and decision makers and government policy makers to ascertain which barriers and facilitators are consistent across cohorts. Using the TDF, seven dominant themes emerged across cohorts: ‘environment context and resources’, ‘knowledge’, ‘social/professional role and identity’, ‘skills’, ‘memory attention and decision-making processes’, ‘emotion’ and ‘social influence’.

### Mapping barriers and facilitators to intervention functions

Using the TDF, BCW and TATT [36, 45, 46], intervention functions were mapped to the seven dominant themes identified in study 2. The resulting list of intervention functions with corresponding BCTs was then mapped to each specific barrier and facilitator [45]. Following these steps, one researcher (A.W.) created nine evidence-based intervention options to be the basis for co-design exercises with participants of the second stakeholder workshop.

### Stakeholder workshop 2: co-designing interventions

The aim of the second workshop was to co-design interventions to increase the use of SDM in maternity care for a specific decision point. At the start of the workshop, participants voted on which of the two decision points identified in workshop 1 was most appropriate and practical to design interventions to increase the use of SDM in maternity services at the Women’s. The decision point was used to design all interventions.

After selecting and discussing the decision point, workshop participants were asked to prioritize the nine intervention options (supplementary material). Implementation and other practical considerations were considered using APEASE criteria (Affordability, Practicability, Effectiveness and cost-effectiveness, Acceptability, Side-effects and safety, Equity) [37]. This involved three small working groups independently rating each intervention option to generate their top three intervention options with the guidance of a facilitator. Participants then adapted the prioritized intervention options to be suitable for implementation at the Women’s on the basis of APEASE. Importantly, although intervention options were put to participants, it was ultimately their choice how to design the interventions.

## Results

### Participants

Eighteen stakeholders participated in either one or both stakeholder workshops (15 in workshop 1, 10 in workshop 2) (Additional file 1). Across the two workshops, participants included six clinicians (obstetricians, midwives and allied health), six health service leaders, three health service administrators, two patients and one government policy maker. Most health service leaders (4/6) were also clinicians. Seven participants attended both workshops (two health service leaders, two health service administrators, two patients and one government policy maker).

### Workshop 1

Multiple decision points were elicited through journey mapping. A final list of nine possible decision points was developed for prioritization:

1. Which model of care to pursue,
2. Type of feeding,
3. Pain relief during labour,
4. Childbirth education,
5. Delivery type,
6. Group B streptococcus (GBS) screening,
7. Screening for genetic or chromosomal conditions,
8. Vaginal birth after caesarean section, and
9. When to be admitted to hospital during labour.

Consistent with the findings of qualitative in-depth interviews, a clear theme emerged regarding decision points which guided subsequent steps in the first workshop – lack of true choice. Participants identified this as a key barrier to implementing an intervention around certain choices. For example, clinicians, health administrators and decision makers were conscious that, due to government funding restrictions, the Women’s was not able to provide all care options for all potential SDM decision points. For example, general practitioner care in the community versus caseload midwife led care at the hospital is not a ‘true’ SDM opportunity because only a limited number of people can access caseload care. This was also true of ‘when to be admitted to hospital during labour’ as this decision is heavily influenced by hospital resourcing (i.e. staff, bed availability).

Therefore, workshop participants eliminated two decision points where no true choice existed. The remaining seven decision points were prioritized considering their appropriateness, feasibility, clinical importance, benefit and acceptability. This resulted in ‘pain relief during labour’ and ‘vaginal birth after caesarean’ as the most promising decision points for intervention design.

### Workshop 2

At the beginning of the second stakeholder workshop, participants unanimously chose ‘pain relief during labour’ as the most appropriate and practical decision point for which to design an intervention over the next 12 months.

During workshop 2 the nine intervention options were discussed and prioritized in small groups (of three to four participants) with a focus on pain relief during labour. Numerous options for pain relief during labour were identified including non-pharmacotherapy (i.e. relaxation, hypnosis, music, bio-feedback) and pharmacotherapy (i.e. epidural, inhaled analgesia) interventions [47]. There are different risks and benefits to each option, and patients will have different analgesic needs.

During the conversation, key themes emerged when applying the APEASE criteria. These themes were agreed on by participants and used to influence their design of interventions (Table 1).

**Table 1:** Key themes from application of APEASE criteria to intervention options

Intervention option example (TDF domain)	Key theme	APEASE criteria
Extend appointment times with patients by 10–15 min AND/OR Add an additional appointment time during pregnancy journey (Environmental context and resources)	It was not practical or acceptable to extend appointment times to support SDM as this would result in resourcing issues and changes in workflow that were too difficult for other systems to absorb	Affordability Acceptability Practicability
Update booking letters to patients to include information about what to expect in terms of SDM while at the Women’s (Knowledge)	Adding additional information about SDM to letters sent to patients advising them on their care at the Women’s (booking letters) was deemed too overwhelming and not suitable by participants	Equity
Update care guidelines to include SDM and how it applies to the specific decision being made by clinician/patient The guideline text may include steps of shared decision-making (Environmental context and resources)	Participants felt changes to care guidelines to include SDM would not be enough to change clinician’s behaviour and would not be effective	Effectiveness
Use feedback to provide clinicians with insights into whether or not they are using SDM and how they might be able to improve This method could use other trained clinicians, when practising or improving clinicians should be provided positive feedback (Social Influence, Knowledge, Skills)	Participants (especially clinicians) felt having clinicians provide feedback to their colleagues about their SDM behaviour in clinic would not be acceptable or practical as this would require additional resources and clinician time	Affordability Acceptability Practicability

The following interventions were co-designed by participants to increase the use of SDM for discussion about pain management. Table 2 provides an overview of how the intervention functions were mapped to the TDF and BCTs.

**Table 2:** Illustrative examples of mapping intervention components to intervention functions based on the TDF

TDF domain (study 2)	Sub-theme from study 2	Intervention function	BCTs identified using the Theory & Techniques Tool	Example from intervention
Knowledge	Patients don’t know their options, risks and benefits	Education	5.1 Information about health consequences	Use online videos to explain options, risks and benefits (intervention 1)
			5.3 Information about social and environmental consequences	Use online video to promote shared record of patient notes (intervention 1)
Skills	Clinicians feel they don’t have the skill or experience to practise SDM but believe experience is the best facilitator of SDM	Training	4.1 Instruction on how to perform the behaviour 6.1 Demonstration of the behaviour 8.1 Behavioural practice and rehearsal	Clinical champions trained in how to have SDM conversation around pain management in labour for different scenarios (intervention 3) Junior clinicians provided clinical education sessions using the simulation lab to practise using SDM for pain management conversations (intervention 3)
Environment, context and resources	Stakeholders believe patients need more time to consider their options	Environmental restructuring	12.5 Adding objects to the environment	Provide access to their record via the patient portal prior to the patient’s first appointment at 18–20 weeks (intervention 1)
			7.1 Prompts/cues	Prompt patients to complete the record using antenatal clinic reception staff (intervention 1) Prompts clinicians to go through the record during their allotted time using the EMR (intervention 1)
Social/professional role and identity	Clinicians believe patients should be involved in decisions about their care, but are uncertain whether their colleagues agree	Persuasion	9.1 Credible source 6.3 Information about others’ approval	Communications campaign raising awareness of other clinicians agreeing with and using SDM in practice (Intervention 2)
	Clinicians feel supported to practise SDM when there is a clear mission statement promoting SDM	Persuasion	9.1 Credible source 6.3 Information about others’ approval	Communications campaign raising awareness of health service’s commitment to SDM (intervention 2)
	Patients believe they should be included in decisions about their care	Persuasion	9.1 Credible source	Communications campaign raising awareness of SDM and pain management options (intervention 2)
	Stakeholders believe junior clinicians are not provided adequate role modelling for SDM	Modelling	6.1 Demonstration of the behaviour 6.2 Social comparison	Clinical champions promoted to the cohort as being able to assist others practising SDM and answering questions (intervention 3) Junior clinicians provided a tool to observe instances of SDM they see in practice (intervention 3)

Table 2 shows each TDF domain, related sub-theme and intervention function. BCTs were chosen using the Theory & Techniques Tool (TATT) by mapping each TDF domain to BCTs that have known links

TDF, theoretical domains framework; BCT, behavioural change technique

### Intervention 1: environmental restructuring and education

Intervention 1 consists of multiple components using Victorian Government’s maternity record (the record) in consultations between patients and clinicians. It would involve updating online videos to include reference to pain management options, record and SDM; providing access to the record via the patient portal prior to the patient’s first appointment at 18–20 weeks; prompts by antenatal clinic reception staff to complete the record; and electronic medical records (EMR) prompts to clinicians to go through the record during their allotted time. Barriers and facilitators from study 2 included patients, clinicians, health service administrators and decision makers and government policy makers belief that patients should be provided information earlier in their pregnancy to allow them time to think about their decision options. Furthermore, clinicians find it easier to have SDM conversations with patients who have knowledge of their options. These barriers and facilitators relate to ‘knowledge’ and ‘environmental context and resources’ in the TDF [31]. The intervention functions mapped to these TDF domains were ‘environment restructuring’ and ‘education’. The intervention would require updating the Victorian Government’s maternity record (the record) to be included on the Women’s EMR and patient portal to allow access by both clinicians and patients.

### Intervention 2: persuasion

Intervention 2 is a multi-layered persuasive communications campaign designed to promote the use of SDM with regard to pain management. It is a dual-audience campaign that targets patients and clinicians. Patients would be targeted with the intention of increasing their awareness of their choice of options for pain management during labour. Clinicians would be targeted with the intention of persuading them to use SDM with patients to discuss and decide on pain management options by showing other clinicians agreeing with and performing SDM in practice. Numerous communication channels would be used including the Women’s intranet, email, posters around the hospital, SMS prompts and booking letters. Specific barriers and facilitators addressed (from study 2) include clinicians’ belief that patients should be involved in decisions about their care, but that they are uncertain whether their colleagues agree. Clinicians feel supported to practise SDM when there is a clear mission statement promoting SDM. Patients believe they should be included in decisions about their care and believe clinicians should provide recommendations (and not only provide information). These barriers and facilitators are related to ‘social/professional role and identity’ on the TDF [31] and map to the intervention function ‘persuasion’.

### Intervention 3: modelling and training

Intervention 3 is made up of two parts: part 1 involves establishing a team of clinical champions to model SDM with regard to pain management throughout their work, and part 2 involves providing clinical education and training to junior clinicians on how to do pain management SDM in practices. In part 1, clinical champions would be trained in how to have SDM conversation around pain management in labour for different scenarios. Once trained, these clinicians would be promoted to the broader cohort of clinicians as clinical champions able to assist others practising SDM and answering questions. In part 2, junior clinicians would be provided clinical education sessions using the simulation lab (an onsite lab used to practise clinical scenarios) to practise using SDM for pain management conversations. They would also be provided a tool to observe instances of SDM they see in practice. Results from study 2 show that clinicians feel they do not have the skills or experience to practise SDM but believe experience is the best facilitator of SDM. Additionally, many clinicians believe other clinicians do not want to practise SDM and junior clinicians lack role modelling of how to do SDM in practice. These barriers and facilitators align to the TDF domains ‘social/professional role and identity’ and ‘skills’ [31]. The intervention functions ‘modelling’ and ‘training’ address the barriers and facilitators.

### Factors for success across all interventions

Throughout the stakeholder workshops, key implementation and other practical considerations from the APEASE criteria emerged for what factors should be included in interventions to increase their chance of success (Table 3).

**Table 3:** Factors for intervention success using the APEASE criteria

APEASE criteria	Factors for interventions to increase success
Acceptability	Cultural factors – specifically alignment with strategic direction, support of leaders across disciplines, and adopting clinical champions to act as role models – were felt to be critical enablers of success. Participants also felt it was crucial to consider both clinicians and patients when highlighting and measuring the benefits to both in terms of patient safety and other outcomes. A further key success factor identified was on-going co-design and co-ownership of interventions by stakeholders across the service
Practicability	For interventions to be practical, participants felt they should work within the current systems of care. Participants spoke about the importance of using the EMR and Patient Portal to provide information to patients, become a communication channel between patients and clinicians and, importantly, have a shared record of SDM Participants felt it most practical to make changes to established ways of working, for example adding prompts to the EMR or adding additional information to informational videos. Any intervention would also need to align with information provided across the health service
Affordability	Participants were sure that interventions would be most affordable to the Women’s if they were able to draw upon existing resources from a mix of different departments. This would allow budget restraints to be shared
Effectiveness	Participants felt that departments having co-ownership of interventions would reduce the burden on any one department’s resources. They felt this would also reduce the siloed nature of implementation projects, which can contribute to failure of implementation Participants also highlighted that the interventions must be strengths based (i.e. highlight what clinicians are doing well, rather than what they are doing wrong or poorly) and aim to increase self-efficacy of clinicians in order to be successful. Messaging should also be consistent across all platforms
Equity and side effects	Participants felt it was important to strike a balance between active (conversational) and passive (information provision) dimensions of SDM. Participants also emphasized the need for equitable access to interventions. For example, any information must be provided in languages other than English, and all intervention items should be provided across different formats (i.e. paper, online, SMS) to ensure equitable access

## Discussion

This is the first known study that integrates co-design and behavioural science to develop SDM interventions in maternity care. Drawing upon previous systematic review and in-depth qualitative studies, three interventions were co-designed by participants over two facilitated multi-stakeholder workshops. The interventions were designed for the decision point prioritized by participants. Participants chose between pain management during labour and VBAC. These decision points are both compatible with SDM as there is a choice available to patients and opportunities to participate in a SDM process. Barriers and facilitators to SDM at the service were incorporated using the TDF, BCW and TATT [36, 45, 46] and mapped to intervention functions.

The first intervention, proving clinician and patient access to the record via the EMR and patient portal and supported by informational videos, focused on ‘environmental restructuring’ and ‘education’. Although not specifically a decision aid, this intervention is very similar (i.e. providing formats through which to share information, and to have a collaborative conversation about pain management options, risks and preferences). There have been promising results for decision aid implementation in maternity care [22, 48]. ‘Education’ is a frequent intervention used in healthcare to change behaviour [49]. Although ‘education’ interventions are also common in SDM, being the basis of 73 of 87 studies included in a Cochrane review [29], only 37% of these had a positive effect. A recent study of SDM in pain management during labour used educational videos to convey information to patients [50]. The results were positive, with patients reporting increased understanding of their options, higher satisfaction with the information received, and the quality of pain relief. ‘Environmental restructuring’ is less commonly used in SDM research [51]. The intervention designed by participants includes restructuring the environment to allow patient questions about pain management to be included on the EMR and patient portal. This is similar to online question prompt lists that aim to increase patient question asking as a way to increase SDM [52].

The second intervention focuses on the intervention function ‘persuasion’ to increase the use SDM in pain management. An example of an intervention using persuasion is ‘Ask3Questions’, a patient-targeted campaign used to increase the awareness of SDM [53]. The campaign was originally developed in Australia by Shepherd et al. (2011) and has been implemented in the United Kingdom alongside the Making Good Decisions in Collaboration (MAGIC) programme. Preliminary results are positive with increased health service awareness and interest in the programme, and positive reactions from patients who are motivated by the campaign which gives ‘permission’ to be involved in decisions about their care.

The third intervention combines ‘modelling’ and ‘feedback’. An example of modelling and feedback is the MAGIC programme, which has showed promising results when clinicians are provided with time and space to practise SDM and receive feedback [54]. The MAGIC team found the ‘Three Talk Model’ [4] to be the most useful tool to communicate, build skills and promote positive attitudes towards SDM. The ‘Three Talk Model’ involves working as a team and describing choices (team talk), discussing the risks and benefits of each option (option talk) and discussing preferences before making a decision (decision talk) [4]. This model could be used by the Women’s during simulation lab exercises to embed the concepts of SDM in different scenarios regarding pain management during labour.

This study has some limitations. For instance, although patients were included, the ratio of patients to clinicians was low. Ideally, more patients would have participated; however, those that signed up were unable to participate for a number of reasons, including coronavirus disease 2019 (COVID-19) and changed work schedules. Likewise, having a greater number of participants participate in both workshops would have been ideal to build knowledge. Due to COVID-19, the health service faced significant staffing shortages and many participants from the first study were unable to attend due to work demands. However, an advantage of this disruption was cross-validation of ideas between the workshops.

A major strength of this study is the Women’s ongoing commitment to research in this space. Senior leaders allowed clinicians and staff to be involved in the research during an incredibly difficult time for the service with COVID-19. This study included stakeholders in addition to patients and clinicians [23, 24]. These stakeholders have visibility of, and influence on, organizational and system-level factors critical to the success of SDM interventions in this setting. For example, they were able to providing insight into the funding mechanisms and resource limits underlying the way in which options are presented leading to a ‘lack of true choice’. This is an important distinction in SDM policy and practice and was also observed in study 2. Although SDM policy promotes involvement in decision-making for patients, there are organizational and system-level factors that prevent health services from including patients in decisions about their care, the most pertinent example being choice of care model. There is robust evidence for the benefits of continuity of care models [55, 56]; however, there is limited funding available for health services, with only 8% of patients funded [57].

Further strengths of this study include a strong co-design focus, addressing a lack of stakeholder engagement in previous interventions aiming to educate patients on their options, risks and benefits [50, 58] and use of behavioural science theory to underpin intervention development, which is rare in maternity care SDM intervention development [25, 26, 59]. The use of the TDF, BCW and TATT ensured co-designed interventions were based on theory and evidence while being appropriate for the specific context in which they would be employed [32], thus increasing their chance of success compared with interventions designed without theory and evidence [26, 29, 30].

Specifically, the BCW provides a framework for developing interventions to change behaviour; however, as Faija et al. [38] report, it does not provide explicit detail on how decisions are made between and within steps. This study addresses this gap by providing explicit detail of each step in the BCW and the decisions made between each step. Furthermore, this research provides detailed steps of the co-design methods used to prioritize the decision point of interest (pain relief during labour), and potential interventions.

By integrating behavioural science and co-design methods, many of which overlap, this study has optimized the chance of success of the developed interventions. An important next step is to pilot test the effectiveness of intervention(s) developed. Testing interventions would most likely require changes based on the stakeholder involved, for example what works for some groups of midwives may not work for other. This would provide data on the extent to which the interventions were optimized by this development approach compared with methods that do not employ theory or co-design.

## Conclusion

This study, based on formative review and qualitative research and drawing upon established behavioural science theory and techniques, co-designed three interventions to increase the use of SDM around pain management in maternity care in a large Victorian hospital. Intervention 1 includes patient and clinician access to the Victorian Government’s maternity record (the record) via the patient portal and EMR for use in consultations and supported by informational videos. Intervention 2 includes a multi-layered persuasive communications campaign designed to promote the use of SDM with regard to pain management. The third intervention involves establishing a team of clinical champions to model SDM with regard to pain management throughout their work and providing clinical education and training to junior clinicians on how to do pain management SDM in practice. These interventions addressed barriers and facilitators using the behaviour change techniques of ‘environmental restructuring’, ‘education’, ‘persuasion’, ‘modelling’ and feedback’. This study addressed gaps in the literature by involving end users and stakeholders throughout the co-design process. In detailing how users were involved meaningfully and materially and how key decisions were made, the study provides guidance to health services grappling with the transition from policy to practise in the critical area of pain control during labour.

## Supplementary Information

**Additional file 1.** Supplementary Materials.

## Data Availability

The datasets are available from the corresponding author on reasonable request.
