# Supplementary material for: Co-designing a theory-informed intervention to increase shared decision-making in maternity care
Source: Health Res Policy Syst. 2023 Jan 31;21:15. doi: 10.1186/s12961-023-00959-x (PMC9888748; doi:10.1186/s12961-023-00959-x)
Supplement: Supplementary file 1 — Additional file 1. Supplementary Materials. [file 12961_2023_959_MOESM1_ESM.docx]

**Supplementary Materials**

1. Inclusion and Exclusion Criteria of Participants
2. Sampling method for recruitment of clinicians, health service administrators, health service decision makers and government policy makers
3. Characteristics of Study Participants Across Workshop 1 and 2
4. Workshop Prompts
5. Intervention Options
6. The Consolidated Criteria for Reporting Qualitative Studies (COREQ): 32-item checklist
7. **Inclusion and Exclusion Criteria of Participants**

Participants were eligible for inclusion in this study if they met the following criteria:

Patients:

- Had been involved in or expressed interest in being involved in the qualitative interviews of the previous study
  - Inclusion criteria for the previous study:
    - Patients who had given birth in the previous nine months at the Royal Women’s Hospital
    - Were eligible participants for the Victorian Health Experience Survey (VHES)
  - Exclusion criteria for VHES:
    - Deceased Patients
    - Inpatient and Maternity Episodes and Emergency Attendances involving Termination of Pregnancy and Perinatal Death
    - Maternity Episodes without a live birth (vaginal or caesarean)
    - Patient care types for Palliative Care, Posthumous Organ Procurement, Unqualified Newborn and Maintenance Care
    - Inpatient and Maternity Episodes with a VAED Separation Referral to community palliative support
    - Patients with a Preferred Language that is not one of the 16 nominated Community Languages
    - Inpatient and Maternity Inpatients not discharged to a private residence/accommodation or who did not leave against medical advice
    - Maternity Inpatients where any associated baby inpatient episode is not discharged to a private residence/accommodation or who did not leave against medical advice
    - Inpatient and Maternity Inpatients that opt for exclusion

Clinicians, Health Service Administrators and Decision Makers:

- Were eligible if they worked with or in the Maternity Care Department at the Royal Women’s Hospital

Government Policy Makers:

- Were eligible if they were current Victorian Government employees and had experience with SDM implementation

1. **Sampling method for recruitment of clinicians, health service administrators, health service decision makers and government policy makers**

Health service staff (clinicians, health service administrators and decision makers) and government policy makers were selected using snowball sampling (1). Participants who were involved in the previous qualitative interviews were invited to take part in the workshops. If they were unable to attend they were asked to provide up to three names of participants in their cohort who were at a similar seniority level and/or practice area to themselves.

1. **Characteristics of Study Participants Across Workshop 1 and 2**

| **Clinicians** | 7* |
| --- | --- |
| Midwives | 4* |
| Obstetricians | 2 |
| Allied Health | 1 |
|  |  |
| **Health service** | 8* |
| Health service administrators | 3 |
| Health service leaders / decision makers | 5* |
|  |  |
| **Patients** | 2 |
|  |  |
| **Government Policy Makers (PM)** | 1 |

*some health service staff members have multiple roles across clinical, administration, and management. They were asked to respond from the perspective of their predominant role.

**Workshop Prompts**

**Workshop 1**

- Tell us a story about how you came to be passionate about maternity – where did it begin?
- What does it mean to have an exceptional patient experience the Women’s?
- Journey mapping – what are the decision points faced by women experiencing care at the Women’s?
- What decisions are there?
  - What are the options? Is do nothing an option?
  - When does the decision have to be made by? i.e. when is the cut-off point?
  - When could the decision be presented? – could this be earlier than when the decision needs to be made?
  - Who is involved in the decision? Is there anyone else? Think about support people, administrators, etc.
  - Where could the conversation happen?
- Score the decision points based on a range of factors (feasibility, clinical importance, patient benefit, clinician acceptability).
  - What are your top two decision points based solely on feasibility (i.e. what decision points are more likely to be implemented)?
  - What are your top two decision points based solely on clinical importance?
  - What are your top two decision points based solely on patient benefit?
  - What are your top two decision points based solely on clinician acceptability?

**Workshop 2**

- Using the intervention ideas provided please prioritise interventions based on APEASE and decide your top three
  - **Acceptability**
    - Is the intervention acceptable to key stakeholders? This includes the target group, potential funders, practitioners delivering the interventions and other relevant groups.
  - **Practicability**
    - Can it be implemented at scale within the intended context, material and human resources?
    - What would need to be done to ensure that the resources and personnel were in place, and is the intervention sustainable?
  - **Effectiveness**
    - How effective is the intervention in achieving Shared Decision Making?
    - Will it reach the intended target group and how large an effect will it have on those who are reached?
  - **Affordability**
    - Can the necessary budget be found for it?
    - Will it provide a good return on investment?
  - **Side-effects**
    - What are the chances that it will lead to unintended adverse or beneficial outcomes?
  - **Equity**
    - How far will it increase or decrease differences between advantaged and disadvantaged sectors of society?
    - For each intervention, work in groups to give a Yes, No, or unsure to each intervention.
- Each group given an intervention to use as a basis to design how the intervention could be implemented using APEASE and mode of delivery considerations.

1. **Intervention Options**

**Option 1**

Modelling: Senior Clinicians demonstration to junior clinicians how to have an SDM interaction with a patient via role-play exercise.

**Option 2**

Restructure the environment: Informative videos could be sent to patients prior to their appointment or provided on iPads in the waiting room AND

Provide a form to patients so they can note down question for the clinician.

**Option 3**

Restructure the environment: Update care guidelines to include SDM and how it applies to the specific decision being made by clinician/patient

The guideline text may include steps of shared decision making

**Option 4**

Persuasion: Use a multi-layered communications campaign to promote the use of SDM by clinicians at the Women’s to clinicians, administrators and decision makers.

AND use a campaign targeted at patients

**Option 5**

Feedback on behaviour: Use feedback to provide clinicians with insights into whether or not they are using SDM and how they might be able to improve.

This method could use other trained clinicians, when practicing or improving clinicians should be provided positive feedback

**Option 6**

Persuasion and Education: Update booking letters to patients to include information about what to expect in terms of SDM while at the Women's.

**Option 7**

Environmental Restructuring: Extend appointment times with patients by 10-15 minutes

AND/OR Add an additional appointment time during pregnancy journey

**Option 8**

Environmental Restructuring: Use Electronic Medical Record to prompt clinicians to use SDM

**Option 9**

Environmental Restructuring: Use Electronic Medical Record that clinician's fill in during the appointment that both clinician and patient has access to via the Patient Portal

1. **COREQ checklist**

**The Consolidated Criteria for Reporting Qualitative Studies (COREQ): 32-item checklist**

Developed from: Tong A, Sainsbury P, Craig J. Consolidated criteria for reporting qualitative research (COREQ): a 32-item checklist for interviews and focus groups. International Journal for Quality in Health Care. 2007. Volume 19, Number 6: pp. 349 – 357

| **No. Item** | **Guide questions/description** | **Page reported on**  **Notes** |
| --- | --- | --- |
| **Domain 1: Research team and reﬂexivity** | | |
| ***Personal Characteristics*** | |  |
| 1. Inter viewer/facilitator | Which author/s conducted the interview or focus group? | Title page |
| 2. Credentials | What were the researcher’s credentials? E.g. PhD, MD | Title page |
| 3. Occupation | What was their occupation at the time of the study? | Title page |
| 4. Gender | Was the researcher male or female? | The researchers were female (5) and male (1) |
| 5. Experience and training | What experience or training did the researcher(s) have? | This study was conducted by qualitatively trained researchers with experience in behavioural science, implementation science and/or psychology and public health. |
| ***Relationship with participants*** | | |
| 6. Relationship established | Was a relationship established prior to study commencement? | Two researchers (AW, LS) had a prior relationship with two participants through previous work. None of the other researchers were known to participants prior to the workshops. |
| 7. Participant knowledge of the interviewer | What did the participants know about the researcher? e.g. personal goals, reasons for doing the research | Participants were informed that the researchers were interested in co-designing interventions for SDM at the Women’s. |
| 8. Interviewer characteristics | What characteristics were reported about the inter viewer/facilitator? e.g. Bias, assumptions, reasons and interests in the research topic | Participants were informed that the researchers were interested in co-designing interventions for SDM at the Women’s. |
| **Domain 2: Study design** | | |
| ***Theoretical framework*** | | |
| 9. Methodological orientation and Theory | What methodological orientation was stated to underpin the study? e.g. grounded theory, discourse analysis, ethnography, phenomenology, content analysis | Phenomenology was used to underpin the study. An important aspect of this involved the researchers bracketing their own understanding and ideas of maternity care prior to the interviews. We aimed to elicit and describe the structures and core experiences of the participants in order to co-design interventions that would suit the way in which SDM could happen at the Women’s. |
| ***Participant selection*** | | |
| 10. Sampling | How were participants selected? e.g. purposive, convenience, consecutive, snowball | 5 |
| 11. Method of approach | How were participants approached? e.g. face-to-face, telephone, mail, email | 5 |
| 12. Sample size | How many participants were in the study? | 5 |
| 13. Non-participation | How many people refused to participate or dropped out? Reasons? | 5 – Some participants were unable to participate in both workshops due to clinical and staff resourcing demands of the Women’s. |
| ***Setting*** |  |  |
| 14. Setting of data collection | Where was the data collected? e.g. home, clinic, workplace | 5 |
| 15. Presence of non-participants | Was anyone else present besides the participants and researchers? | 5 |
| 16. Description of sample | What are the important characteristics of the sample? e.g. demographic data, date | 5, Supplementary Material |
| ***Data collection*** |  |  |
| 17. Interview guide | Were questions, prompts, guides provided by the authors? Was it pilot tested? | Supplementary Material |
| 18. Repeat interviews | Were repeat inter views carried out? If yes, how many? | N/A |
| 19. Audio/visual recording | Did the research use audio or visual recording to collect the data? | 5 |
| 20. Field notes | Were ﬁeld notes made during and/or after the interview or focus group? | 5 |
| 21. Duration | What was the duration of the interviews or focus group? | Workshop 1: 4 hours with a 30-minute, and two 5-minute break  Workshop 2: 3 hours with a 30-minute break, and two 5-minute breaks |
| 22. Data saturation | Was data saturation discussed? | N/A |
| 23. Transcripts returned | Were transcripts returned to participants for comment and/or correction? | N/A Transcripts were not produced as the workshops were not audio recorded |
| **Domain 3: Analysis and findings** | | |
| ***Data analysis*** | | |
| 24. Number of data coders | How many data coders coded the data? | One researcher coded the entire dataset two other researchers double checked all codes |
| 25. Description of the coding tree | Did authors provide a description of the coding tree? | Themes and subthemes are clearly labelled throughout the manuscript. |
| 26. Derivation of themes | Were themes identified in advance or derived from the data? | Themes were derived from the data. |
| 27. Software | What software, if applicable, was used to manage the data? | NVivo was used to manage the data. |
| 28. Participant checking | Did participants provide feedback on the findings? | Participants did not provide feedback on the findings after workshop 2. |
| ***Reporting*** | | |
| 29. Quotations presented | Were participant quotations presented to illustrate the themes/ findings? Was each quotation identified? E.g. participant number | N/A |
| 30. Data and findings consistent | Was there consistency between the data presented and the findings? | N/A |
| 31. Clarity of major themes | Were major themes clearly presented in the findings? | 6-12 |
| 32. Clarity of minor themes | Is there a description of diverse cases or discussion of minor themes? | Yes, cohort specific minor themes and diverse cases are discussed throughout the results and discussion |

**References:**

1. Creswell JW, Poth CN. Qualitative Inquiry and Research Design: Choosing Among Five Approaches - John W. Creswell, Cheryl N. Poth -. Sage Publications. 2017.
